# Bird Life in Macedonia

**Published:** 1917-07-14

**Authors:** 


### BIRD LIFE IN MACEDONIA.

By AN OFFICER OF THE R.A.M.C., AND THE AUTHOR OF "NATURE NOTES"

Spring came suddenly on Macedonia. There had been icy-cold north-east winds, snow, frost, and bone-penetrating damp till a day, about mid-March, on which the wind veered south, warm sunshine dispersed the clouds, and trees began to show buds. The last half of March was perfect weather, making up for the horribly damp, windy cold of the winter. Immediately with the change the storks came back. I saw the first one on the 12th of March, and on the 18th I saw a flock of nine or ten. It flew to a village of the valley in which are old nests. There the birds spent half an hour or more circling round and round in the air above the houses and trees, and then made off. Close to the village a kite balloon was in the air, but it did not dismay them; a few days later they returned and commenced to repair their nests. About the same time I saw other storks near the nests that are scattered in nearly every village of the valley. All did not arrive together; they drifted in over a period of several days. The birds seemed to be paired on arrival, for I saw no fighting for mates nor love-chases. The rumble and roar of artillery and the constant passage of airplanes seem to disturb birds little if at all. I am curious to see how the storks will behave if a great battle is fought here, and shells hum over their nests and burst above them.

There has been much movement of birds during the month of March. The geese have gone; most of the ducks have gone, but a few are paired and seem to mean to rest here. The only species of duck I have seen paired here is the mallard. Numbers of warblers and willow wrens have arrived, but I cannot give the species, and swallows, hoopoes, cuckoos, redstarts, wheatears, wagtails, and a host of others. The great flocks of rooks and starlings that were conspicuous in the winter have dispersed or gone. I felt sure the rooks were migrants because I did not see rookeries in the country nor did I notice any rooks in it until autumn. In the fourth week of March there were still many rooks in the valley, though the number was greatly diminished, but on March 25 I rode far in the valley and did not see one, nor have I seen one since—English rooks have eggs by the end of March. The rooks that remain here till the end of March must lay later. This may give a clue to their nesting district. A large number of the hawks that frequented the valley this winter have gone away, but numbers remain, many of them paired. I greatly regret that I have no book that would enable me to identify the varieties I see. Several pairs of kites work in my area. It is a most beautiful sight to see them wheeling on the wind and beating their chosen ground. They gradually drift down wind, working to and fro across the wind as they go, holding themselves at the right plane by movements of their forked tails, occasionally giving a stroke of their spread wings. After a long drift they beat up wind again, and again drift down.

There are many kestrels. A few days ago one attracted my attention by its cries. It was wheeling in the air near my tent, swiftly and excitedly circling on extended wings and crying at intervals. It had prey in its claws. The bird wheeled and soared till it reached so great a height, at a distance of some 600 yards from me, that I could scarcely discern it. Suddenly it began to descend at uncommon velocity, planed down over a distance of perhaps half a mile, alighted on a stone a furlong from me, and began to devour that it had been carrying. I thought perhaps its crying and soaring were intended to bring its mate to share its meal.

There is a ruddy-coloured hawk, large and with a strikingly white head. I believe I recollect a picture of a kind of harrier that this bird might be. I frequently see pairs near certain marshy grounds. I hoped they were going to nest here, but I fear certain movements of troops have decided them to seek a quieter country. I have found the nest of a species of eagle. I first saw it in the middle of March just before the storks came. Against the sky I saw a great nest in a tree a long distance from me, and by it a great bird sitting. I knew there were storks' nests in some trees near by, and thought at first it was a stork just arrived and visiting its nest. I rode nearer, and then saw the bird was a species of the eagle tribe. It rose from by the nest when I got near and flew lazily to another near-by tree. I was then aware of another bird of the same kind flying towards me across the marshy ground. It settled by its mate. I saw that the nest was a new nest, a great pile of sticks. A few days later I saw one of the pair sitting on it.

The kestrels are nesting in the roofs of the houses in nearly every village. They compete for sites with the jackdaws. Squabbles are common between the two species, but on the whole relations are friendly. I have seen a pair of kestrels, a pair of turtle-doves, and a couple of pairs of jackdaws sitting amicably on the same roof. It is a daily event to see kestrels and jackdaws on the same house. The birds are astonishingly tame. Though most of the houses are only one storey and so low that sitting on my horse my head may reach above the eaves, the birds will allow me to watch them from a distance of only a few yards. The number of kestrels is surprising. It is common to see ten or a dozen pairs in one village. Their ways are most interesting. At evening time they seem to play in the air before they roost. Then you may see fifteen or twenty or even more circling about together, rising to great heights and wheeling round and round as if in a dance. Then one or more will swoop down at great speed; a most beautiful and graceful descent. Some after this descend, alight on tree or house top, and remain; others rapidly reascend and join again the whirling throng.

At mid-day it is common to see the kestrels hawking over villages with a great company of other birds; swallows, starlings, and jackdaws. Then it is probable they are pursuing insects of some kind, for they double and twist and rise and fall and grab with their claws and often transfer something to their beaks from their claws by bending down the head as they fly. I have not discovered what it is they are preying on at these times—I suppose a kind of beetle. I often see these little hawks eating lizards and mice, but I have never seen one take a bird. I notice that the small birds do not regard them with fear. It is very different, though, when a peregrine falcon or other of the fiercer hawks comes on the scene. I was watching the kestrels in a village east of the Struma a few days ago, early in April, when suddenly a peregrine falcon came gliding swiftly, silently, low down over the roofs of the village. Clamour and cries of alarm rose from many kinds of birds—jackdaws, starlings, finches, buntings, sparrows. The falcon glided into a thorn bush in which were three buntings that perhaps he had marked for prey. But the thick thorns baulked him. He sat in the bush separated only by inches from the little birds. They kept their position in the safe shelter of the bush, seemingly conscious of security, whilst round and about other birds collected and abused the falcon in their respective tongues. After a short while the falcon left the bush and perched upon a tree near by. Then the jackdaws collected in force and mobbed it, but kept at a discreet distance.

Another day I saw a smaller hawk swiftly pursuing a starling that fled with shrieks and sought refuge against my horse, a manœuvre that disconcerted the hawk, so that it swerved and, passing close behind my back, gave up the chase.

Rooks, jackdaws, and crows all commit highway robberies on kites and kestrels. I often watch them chasing the hawks. I think it is only when the hawk is carrying prey that they set upon it. Unencumbered, the hawk would soon shake off such pursuers, or perhaps take counter-offensive with beak and claw, though probably the strong, straight, stabbing beak of the crow tribe is a more efficient weapon than the curved, tearing beak of the hawk. The end of chases often is that the hawk drops something it is carrying, and the rooks descend to seize it. In their turn the rooks are robbed by a kind of seagull—I think the common black-headed gull that haunts the Thames in wintertime. A number of these birds arrived in the Struma valley during the winter, and remained for several weeks, especially frequenting a wide branch valley down which a tributary stream crawls, and in which are camps and collections of stores; here they shared refuse with rooks and crows. It was common to see one or more gulls chasing a rook that had seized a tasty morsel. The gulls were the faster in flight, but the rook is a master of the art of evasion, so often the chases lasted for minutes and extended for miles of devious course. The gulls always secured the loot unless the rook could make the harbour of a tree. Such refuge attained, the gulls gave up pursuit. Webbed feet are ill-adapted for tree warfare.

During a visit to Salonika in the last days of March I was interested to see flocks of these gulls apparently pursuing and catching insects in the air above the flat lands by the Monastir road. The movements and motions of the birds were similar to those of swallows hawking over English meadows. I could imagine no other reason than pursuit of insects for the activities of these flocks, but I have no idea what species of insect it could be. It is curious that in this country such birds as seagulls, hawks, and crows should hawk insects in the air, but I have no doubt in my mind that all do do so, and so far as I can make out it is often a quite small insect they pursue. In India I have seen all sorts of birds, hawks, crows, and others, pursue and take locusts in the air, but it is certainly no such large insect the birds here are frequently pursuing.

